# Superiority of pulmonary administration of mepenzolate bromide over other routes as treatment for chronic obstructive pulmonary disease

**DOI:** 10.1038/srep04510

**Published:** 2014-03-28

**Authors:** Ken-Ichiro Tanaka, Shota Kurotsu, Teita Asano, Naoki Yamakawa, Daisuke Kobayashi, Yasunobu Yamashita, Hiroshi Yamazaki, Tomoaki Ishihara, Hiroshi Watanabe, Toru Maruyama, Hidekazu Suzuki, Tohru Mizushima

**Affiliations:** 1 Faculty of Pharmacy, Keio University, Tokyo 105-8512, Japan; 2 Faculty of Life Sciences, Kumamoto University, Kumamoto 862-0973, Japan; 3 Department of Internal Medicine, Keio University School of Medicine, Tokyo 160-8582, Japan

## Abstract

We recently proposed that mepenzolate bromide (mepenzolate) would be therapeutically effective against chronic obstructive pulmonary disease (COPD) due to its both anti-inflammatory and bronchodilatory activities. In this study, we examined the benefits and adverse effects associated with different routes of mepenzolate administration in mice. Oral administration of mepenzolate caused not only bronchodilation but also decreased the severity of elastase-induced pulmonary emphysema; however, compared with the intratracheal route of administration, about 5000 times higher dose was required to achieve this effect. Intravenously or intrarectally administered mepenzolate also showed these pharmacological effects. The intratracheal route of mepenzolate administration, but not other routes, resulted in protective effects against elastase-induced pulmonary damage and bronchodilation at a much lower dose than that which affected defecation and heart rate. These results suggest that the pulmonary route of mepenzolate administration may be superior to other routes (oral, intravenous or intrarectal) to treat COPD patients.

Chronic obstructive pulmonary disease (COPD) is a serious health problem and the most important etiologic factor of which is cigarette smoke (CS). COPD is currently the fourth leading cause of death in the world and its prevalence and mortality rates are steadily increasing^1^. This disease state is defined by a progressive and not fully reversible airflow limitation associated with an abnormal inflammatory response-mediated permanent enlargement of the pulmonary airspace^1^,^2^,^3^. Thus, for the clinical treatment of COPD, it is important not only to improve the airflow limitation by bronchodilation, but also to suppress disease progression by controlling inflammatory processes.

Bronchodilators (β_2_-agonists and muscarinic antagonists) are currently used for the treatment of COPD owing to their ameliorating effects on airflow limitation^2^,^4^,^5^. Steroids are also used to suppress inflammatory processes in COPD patients; however steroids do not significantly modulate disease progression or mortality^5^,^6^, because the inflammation associated with COPD tends to be resistant to steroid treatment^7^. Thus, the development of new types of anti-inflammatory drugs to treat COPD is paramount.

The number of drugs reaching the marketplace each year is decreasing, mainly due to the unexpected adverse effects of potential drugs being revealed at advanced clinical trial stages. For this reason, we proposed a new strategy for drug discovery and development (drug re-positioning)^8^. In this strategy, compounds with therapeutically beneficial activity are screened from a library of approved medicines to be developed for new indications. The advantage of this approach is that there is a decreased risk for unexpected adverse effects in humans because the safety aspects of these drugs have already been well characterized in humans^8^. From a library of approved medicines, we screened compounds that prevent elastase-induced pulmonary emphysema in mice, and selected mepenzolate bromide (mepenzolate)^9^, which is an orally administered muscarinic receptor antagonist used to treat gastrointestinal disorders (such as peptic ulcers and irritable bowel syndrome)^10^,^11^,^12^. We showed that mepenzolate not only exerts an anti-inflammatory effect via a muscarinic receptor-independent mechanism, but also a bronchodilatory effect via a muscarinic receptor-dependent mechanism^9^.

Oxidative stress, such as superoxide anion, is believed to play a major role in abnormal inflammation in COPD patients and nicotinamide adenine dinucleotide phosphate (NADPH) oxidase plays an important role in the production of superoxide anions^13^. The body contains a number of endogenous anti-oxidant proteins such as superoxide dismutase and glutathione S-transferase, with a decrease in these proteins reported to be involved in the pathogenesis of COPD^14^,^15^. We reported that mepenzolate not only suppressed the elastase-induced production of superoxide anions and NADPH oxidase activation but also stimulated the expression of superoxide dismutase and glutathione S-transferase, suggesting that mepenzolate suppresses elastase-induced pulmonary emphysema via decrease of oxidative stress^9^. Based on these results, we proposed that mepenzolate could serve as a candidate drug for the treatment of COPD.

The route of administration of each particular drug is an important factor to be taken into account when considering its final clinical application. Most muscarinic receptor antagonists currently used for treating COPD patients are administered via the lung^16^ because the systemic administration of this type of drug frequently results in adverse effects on cardiac and intestinal functions (such as arrhythmia, heart palpitations and constipation). In this way, we chose the pulmonary route of mepenzolate administration (intratracheal administration or inhalation) in our previous study on mice^9^. On the other hand, since mepenzolate was approved for use as an orally administered drug, the development of this drug to be taken orally for COPD would be more convenient compared to other administration routes. Thus, to determine the appropriate route of mepenzolate administration for possible use by COPD patients, we examined here the effect of different administration routes on this drug's beneficial and adverse effects in mice. When administered intratracheally, mepenzolate showed protective effects on elastase-induced pulmonary damage at a much lower dose than that which affected fecal pellet output and heart rate. With respect to the other administration routes (oral, intravenous and intrarectal), mepenzolate showed protective and adverse effects at similar doses. These results suggest that the pulmonary administration route for mepenzolate may be superior to other routes to treat COPD patients.

## Results

### Effect of different administration routes of mepenzolate on pulmonary damage and airway resistance

We recently reported that the intratracheal administration or inhalation of mepenzolate suppressed porcine pancreatic elastase (PPE)-induced inflammatory responses, pulmonary emphysema, alteration of lung mechanics, and respiratory dysfunction^9^. As a first step in the present study, we confirmed these effects of intratracheally administered mepenzolate.

**Figure 1:**
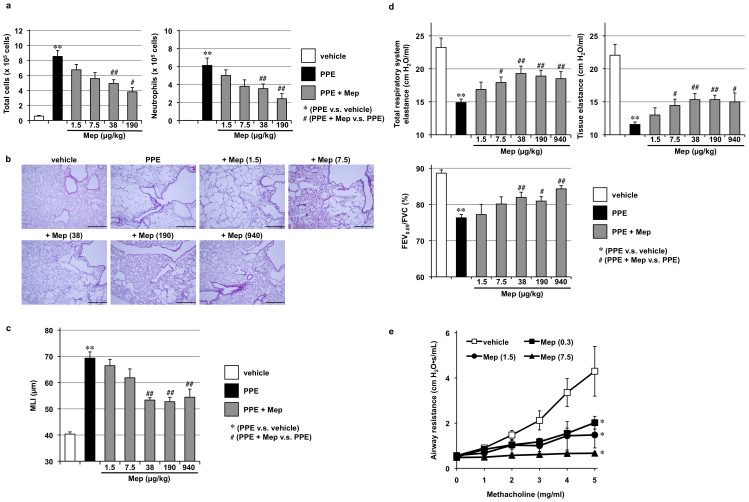
Effect of intratracheal administration of mepenzolate on PPE-induced pulmonary damage and methacholine-induced airway constriction. Mice were treated with or without (vehicle) PPE (15 U/kg) once only on day 0 (a–d). The indicated doses (μg/kg) of mepenzolate (Mep) were administered intratracheally once only (a) or once daily for 12 days (from day 0 to day 11) (b–d). Twenty-four hours after the PPE administration, BALF was prepared and the total cell number and the number of neutrophils were determined as described in the Materials and Methods (a). Sections of pulmonary tissue were prepared on day 14 and subjected to histopathological examination (H & E staining) (scale bar, 500 μm) (b). Airspace size was estimated by determining the MLI as described in the Materials and Methods (c). Total respiratory system elastance, tissue elastance, and FEV_0.05_/FVC were determined on day 14 as described in the Materials and Methods (d). Indicated doses (μg/kg) of mepenzolate (Mep) were administered intratracheally. After 1 h, mice were exposed to nebulized methacholine 5 times and airway resistance was determined after each methacholine challenge as described in the Materials and Methods (e). Values represent mean ± S.E.M. (*n* = 3–10). * or ^#^ *P < 0.05*; ** or ^##^ *P < 0.01*.

As shown in Fig. 1a, the total number of leucocytes and the individual number of neutrophils in bronchoalveolar lavage fluid (BALF), which serve as indicators of pulmonary inflammatory responses, increased after the PPE treatment; this increase was partially suppressed by the simultaneous intratracheal administration of mepenzolate (38 or 190 μg/kg). Histopathological analysis revealed that while PPE administration damaged the alveolar walls and increased mean linear intercept (MLI), this effect could again be partly suppressed by the administration of mepenzolate (38–940 μg/kg; Fig. 1b and c). The alteration of lung mechanics associated with pulmonary emphysema is characterized by a decrease in elastance^17^. PPE treatment decreased both total respiratory system elastance (whole lung elastance, including the bronchi, bronchioles and alveoli) and tissue elastance (elastance of alveoli), both of which were partially restored by simultaneous mepenzolate administration (Fig. 1d). PPE treatment also decreased the FEV_0.05_/FVC ratio (Fig. 1d), which is homologous to the FEV_1_/FVC ratio in humans^18^,^19^. Mepenzolate administration restored the FEV_0.05_/FVC ratio towards control values (Fig. 1d). The bronchodilation activity exerted by mepenzolate was monitored by its inhibitory effect on the increase in airway resistance induced by methacholine^9^. As shown in Fig. 1e, the methacholine-induced increase in airway resistance was completely suppressed by the intratracheal administration of mepenzolate, with the dose required to decrease the airway resistance (0.3 μg/kg) being much lower than that required to protect the pulmonary tissue against PPE-induced damage (38 μg/kg, Fig. 1c). The results in Fig. 1 are thus consistent with those reported previously^9^.

**Figure 2:**
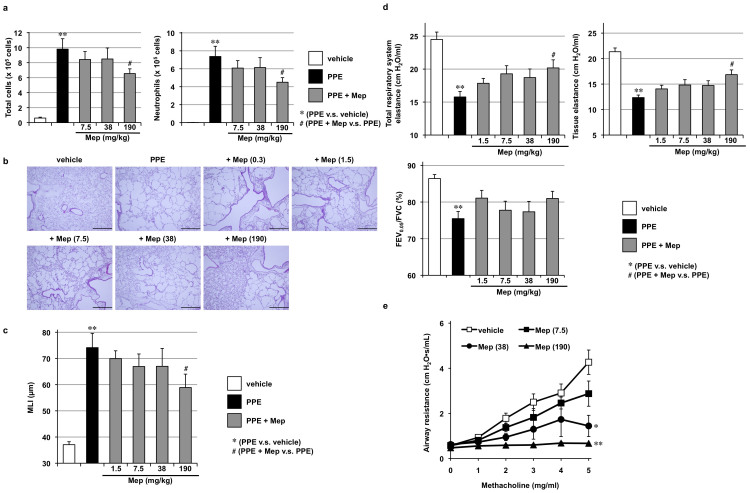
Effect of oral administration of mepenzolate on PPE-induced pulmonary damage and methacholine-induced airway constriction. Administration of PPE, mepenzolate and methacholine was performed as described in the legend of Fig. 1, except that mepenzolate was administered orally (a–e). Analysis of inflammatory responses (a), histopathological examination (scale bar, 500 μm) (b), determination of the MLI (c), measurement of lung mechanics and respiratory function (d) and measurement of airway resistance (e) were carried out as described in the legend of Fig. 1. Values represent mean ± S.E.M. (*n* = 3–8). * or ^#^ *P < 0.05*; ** *P < 0.01*.

We subsequently examined the effects of orally administered mepenzolate on the same parameters as those described above. As shown in Fig. 2a–c, orally administered mepenzolate protected against PPE-induced inflammatory responses and pulmonary emphysema; however, the dose required to achieve this protective effect (190 mg/kg) was much higher than that found when the drug was administered intratracheally (Fig. 1a–c). Orally administered mepenzolate also suppressed PPE-induced alterations of lung mechanics but did not significantly affect respiratory dysfunction (Fig. 2d). The bronchodilatory effect of orally administered mepenzolate was also observed only at higher doses (Fig. 2e) compared with that obtained with intratracheal mepenzolate administration (Fig. 1e). Furthermore, in contrast to the results for intratracheal administration, orally administered mepenzolate showed both bronchodilatory and protective effects against PPE-induced pulmonary disorders at roughly similar doses (Fig. 2).

**Figure 3:**
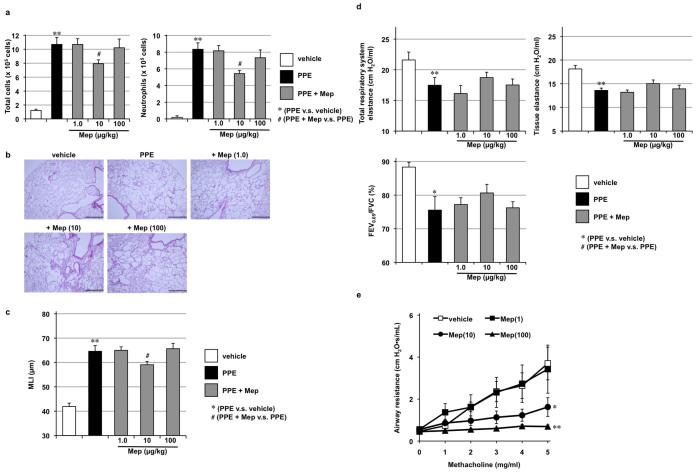
Effect of intravenous administration of mepenzolate on PPE-induced pulmonary damage and methacholine-induced airway constriction. Administration of PPE, mepenzolate and methacholine was performed as described in the legend of Fig. 1, except that mepenzolate was administered intravenously (a–e). Analysis of inflammatory responses (a), histopathological examination (scale bar, 500 μm) (b), determination of the MLI (c), measurement of lung mechanics and respiratory function (d) and measurement of airway resistance (e) were carried out as described in the legend of Fig. 1. Values represent mean ± S.E.M. (*n* = 3–14). * or ^#^ *P < 0.05*; ** *P < 0.01*.

We also examined the effects of intravenously administered mepenzolate. As shown in Fig. 3a–c, this route of mepenzolate administration (10 μg/kg) protected against PPE-induced inflammatory responses and pulmonary emphysema. Compared to the intratracheal administration, although the effective dose was slightly lower via the intravenous route, the extent of amelioration was not as apparent (Fig. 3a–c). Furthermore, intravenous administration of the highest dose of mepenzolate tested for this route (100 μg/kg) did not protect against PPE-induced pulmonary damage (Fig. 3a and c), nor did it significantly restore the lung mechanics and respiratory function, both of which were affected by the PPE treatment (Fig. 3d). These results demonstrate that intravenously administered mepenzolate is not as effective against PPE-induced pulmonary damage as that achieved via the intratracheally administered route. On the other hand, almost complete inhibition of the methacholine-induced increase in airway resistance was observed with the intravenous administration of mepenzolate (Fig. 3e). These results suggest that the protective effects of mepenzolate against PPE-induced pulmonary damage and its bronchodilatory effect are independent of each other.

### Monitoring of the mepenzolate level in blood and tissue after administration of the drug via different routes

**Figure 4:**
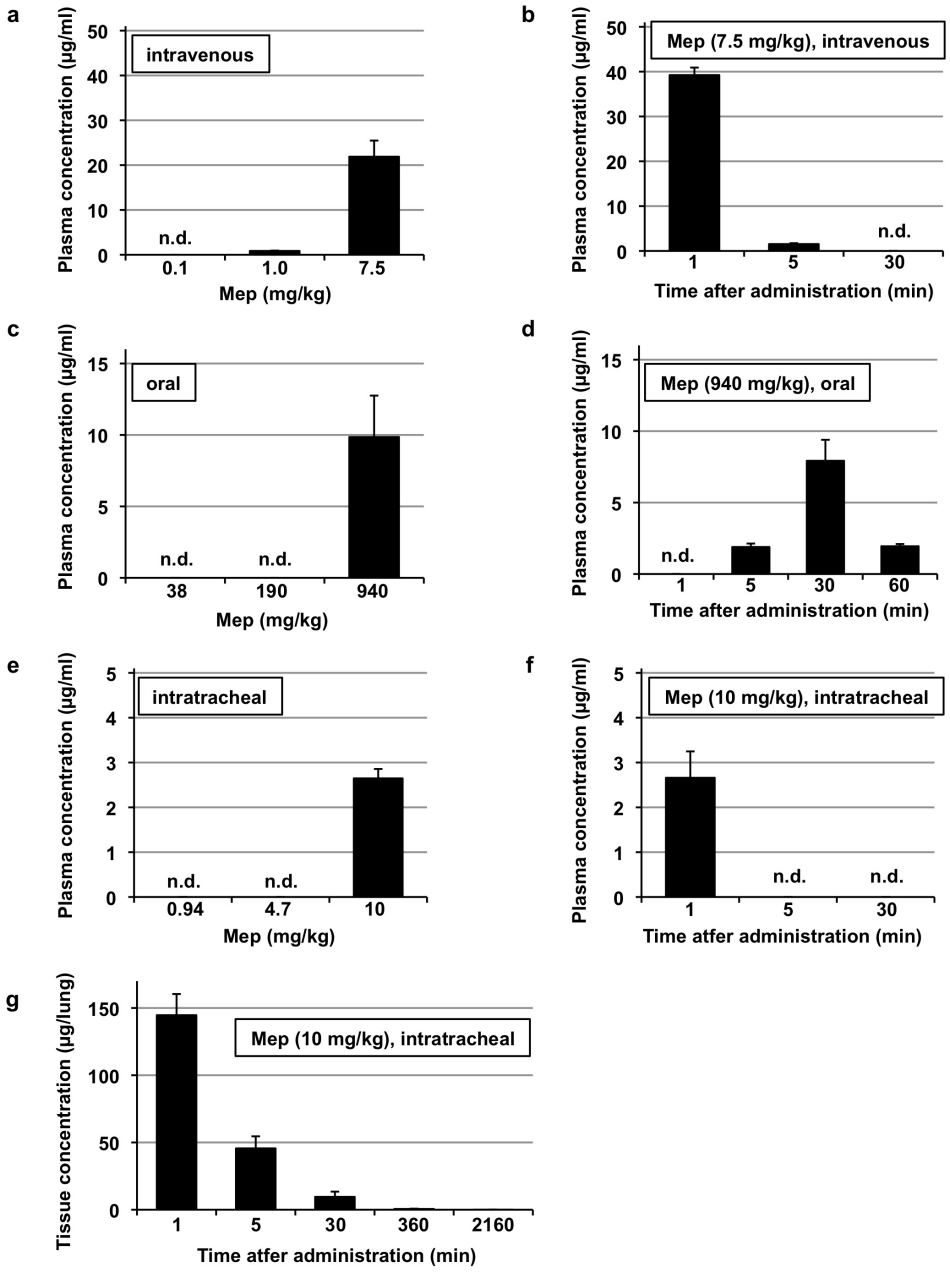
Determination of the level of mepenzolate after administration through various routes. Mice were administered indicated doses of mepenzolate intravenously (a, b), orally (c, d) or intratracheally (e–g). After indicated periods (b, d, f, g), 1 min (a, e) or 30 min (c), blood samples (a–f) or lung homogenates (g) were prepared and the level of mepenzolate was determined as described in the Materials and Methods. Values are mean ± S.E.M. (*n* = 3–4). n.d., not detected.

High performance liquid chromatography (HPLC) analysis was used to determine the level of mepenzolate in plasma and tissue. We initially examined the plasma level of mepenzolate after its intravenous administration, with the detected levels of the drug increasing in a dose-dependent manner (Fig. 4a). Examination of the time-course profile showed that mepenzolate was clearly detectable at 1 min, significantly reduced after 5 min, and undetectable 30 min following its intravenous administration (Fig. 4b), suggesting that mepenzolate is very unstable in blood. We then performed similar analyses to determine plasma mepenzolate levels after oral administration of the drug. As shown in Fig. 4c, mepenzolate could be detected in the plasma only when a very high dose (940 mg/kg) of the drug was administered via this route. Furthermore, the peak level was achieved 30 min after oral administration (Fig. 4d). In contrast, when mepenzolate was administered via the intratracheal route, it could be detected at a relatively lower dose (10 mg/kg) (Fig. 4e). Furthermore, the detection was very rapidly (at 1 min) (Fig. 4f). These results suggests that the efficiency of absorption into the circulation is higher for the intratracheal route of administration than the oral route. We also tried to detect mepenzolate in the lung tissue of treated mice, with the drug detected following administration via the intratracheal route (Fig. 4g), but not for orally or intravenously administered drug (data not shown). The results in Fig. 4g also showed that most of intratracheally administered mepenzolate disappeared from the lung within 30 min.

### Effect of intrarectally administered mepenzolate on pulmonary damage and airway resistance

**Figure 5:**
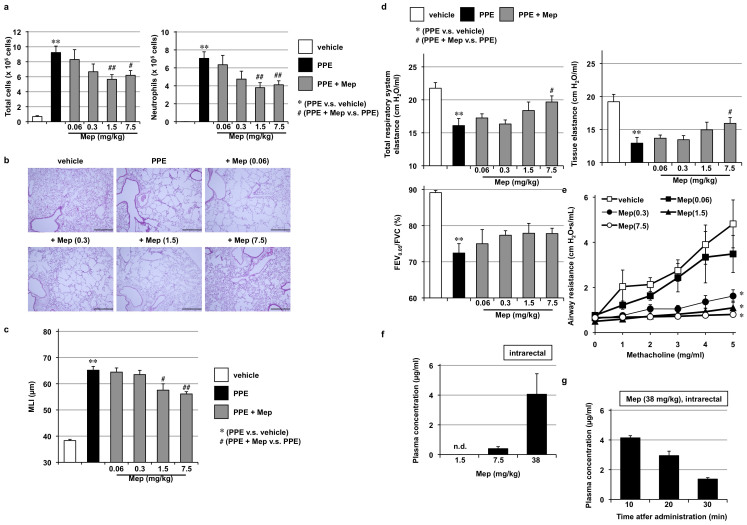
Effect of intrarectal administration of mepenzolate on PPE-induced pulmonary damage and methacholine-induced airway constriction. Administration of PPE, mepenzolate and methacholine was done, as described in the legend of Fig. 1, except that mepenzolate was administered intrarectally (a–e). Analysis of inflammatory responses (a), histopathological examination (scale bar, 500 μm) (b), determination of the MLI (c), measurement of lung mechanics and respiratory function (d) and measurement of airway resistance (e) were carried out as described in the legend of Fig. 1. Mice were administered indicated doses of mepenzolate intrarectally. After 10 min (f) or indicated periods (g), blood samples were taken and the plasma level of mepenzolate was monitored as described in the legend of Fig. 4. Values represent mean ± S.E.M. (*n* = 4–12). * or ^#^ *P < 0.05*; ** or ^##^ *P < 0.01*; n.d., not detected.

It has been reported that, compared to the oral route of administration, the intrarectal route for some drugs results in a much higher uptake efficiency into the circulation due to the circumvention of drug inactivation within the gastrointestinal tract and the first-pass effect, or the higher efficiency of absorption via the rectum compared with the small intestine^20^,^21^. For these reasons, we examined the effect of intrarectally administered mepenzolate on PPE-induced pulmonary damage and airway resistance. As shown in Fig. 5a–c, intrarectally administered mepenzolate showed a protective effect against PPE-induced pulmonary damage at doses of 1.5 or 7.5 mg/kg, which are much lower than that required in the case of oral administration (Fig. 2a–c). Similar results were observed with respect to the PPE-induced alteration of lung mechanics and respiratory dysfunction; however, the amelioration of respiratory function by intrarectally administered mepenzolate was not statistically significant (Fig. 5d). As shown in Fig. 5e, intrarectally administered mepenzolate suppressed the methacholine-induced increase in airway resistance at lower doses to that seen in response to oral administration of the drug (Fig. 2e).

We also determined the plasma level of mepenzolate after the intrarectal administration of this drug. The dose-response and time-course profiles (Fig. 5f and g) revealed that the absorption into the circulation of intrarectally administered mepenzolate is much more efficient and rapid than that seen with orally administered drug (Fig. 4c and d). The results in Fig. 5 thus suggest that the intrarectal route of mepenzolate administration is more effective than the oral route due to the lower effective doses required.

**Figure 6:**
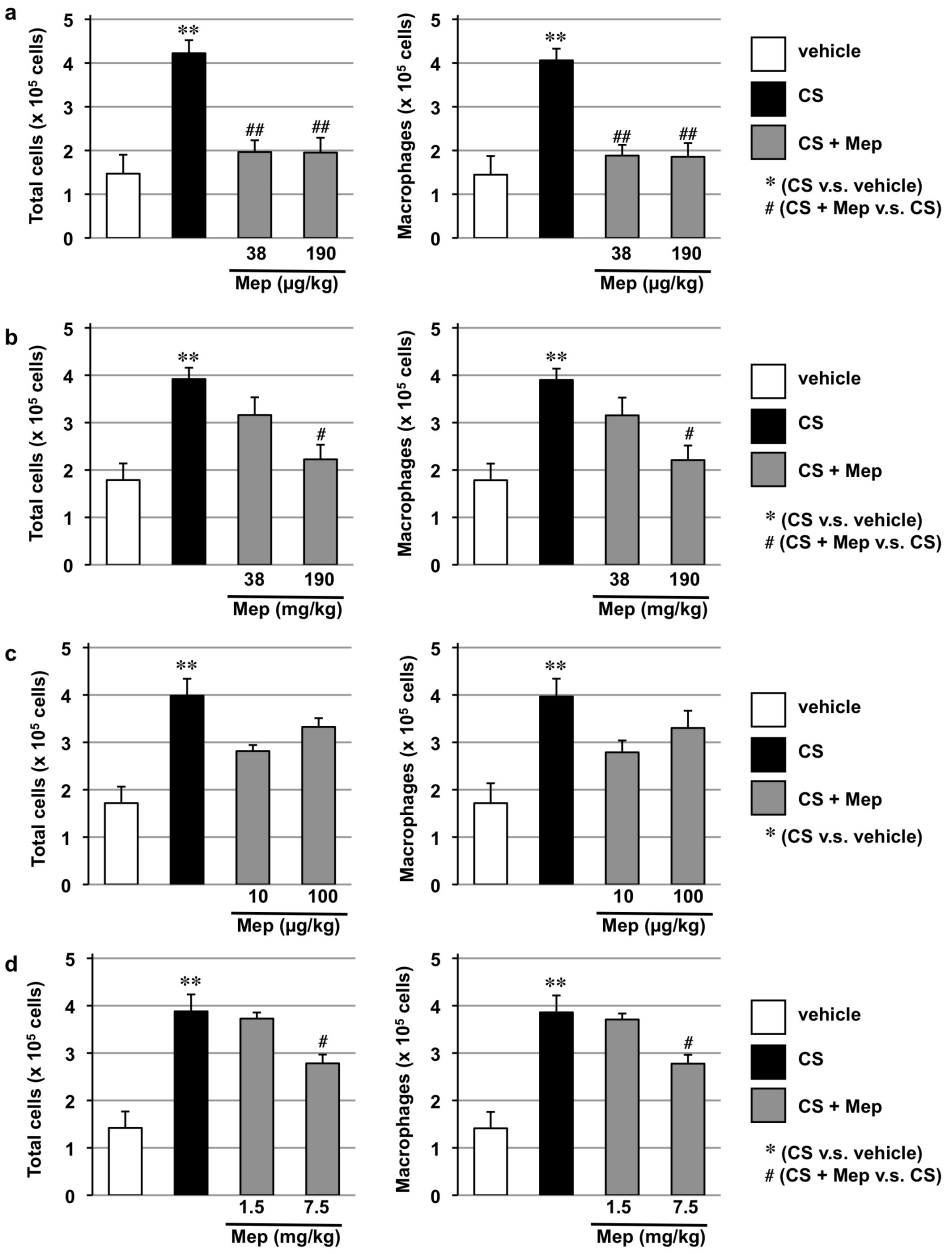
Effect of mepenzolate on CS-induced pulmonary inflammatory responses. Mice were exposed to CS (3 times/day) and intratracheally (a), orally (b), intravenously (c) or intrarectally (d) administered indicated dose of mepenzolate (once daily) for 3 days as described in the Materials and Methods. Six hours after the last CS exposure, BALF was prepared and the total cell number and the number of macrophages were determined as described in the Materials and Methods. Values represent mean ± S.E.M. (*n* = 4–8). * or ^#^ *P < 0.05*; ** or ^##^ *P < 0.01*.

We also examined the effect of different routes of mepenzolate administration on CS-induced lung inflammatory responses. As shown in Fig. 6a, the total number of leucocytes and the individual number of macrophages in BALF increased after the CS treatment and this increase was suppressed by the simultaneous intratracheal administration of mepenzolate (38 or 190 μg/kg). Similar suppression was observed with oral, intravenous or intrarectal administration of mepenzolate (Fig. 6b–d), however, the oral administration required much higher dose of mepenzolate than the intratracheal administration (Fig. 6a, b). Furthermore, the extent of suppression was not so apparent with the intravenous or intrarectal administration as the intratracheal administration and the suppression of increase in the total number of leucocytes and the individual number of macrophages in BALF by intravenous administration of mepenzolate (10 or 100 μg/kg) was not statistically significant (Fig. 6).

### Effect of different administration routes of mepenzolate on the appearance of adverse effects

To determine the appropriate administration route of any drug, it is important to consider not only its beneficial but also its adverse side-effects. For the clinical application of mepenzolate to treat COPD patients, both constipation and arrhythmia (heart palpitations) have been noted as adverse side-effects that occur due to the inhibitory effects of this drug on the muscarinic receptor and the resulting inhibition of intestinal motility and increased heart rate^22^,^23^. We therefore examined the effect of different routes of mepenzolate administration on defecation and heart rate in treated mice.

**Figure 7:**
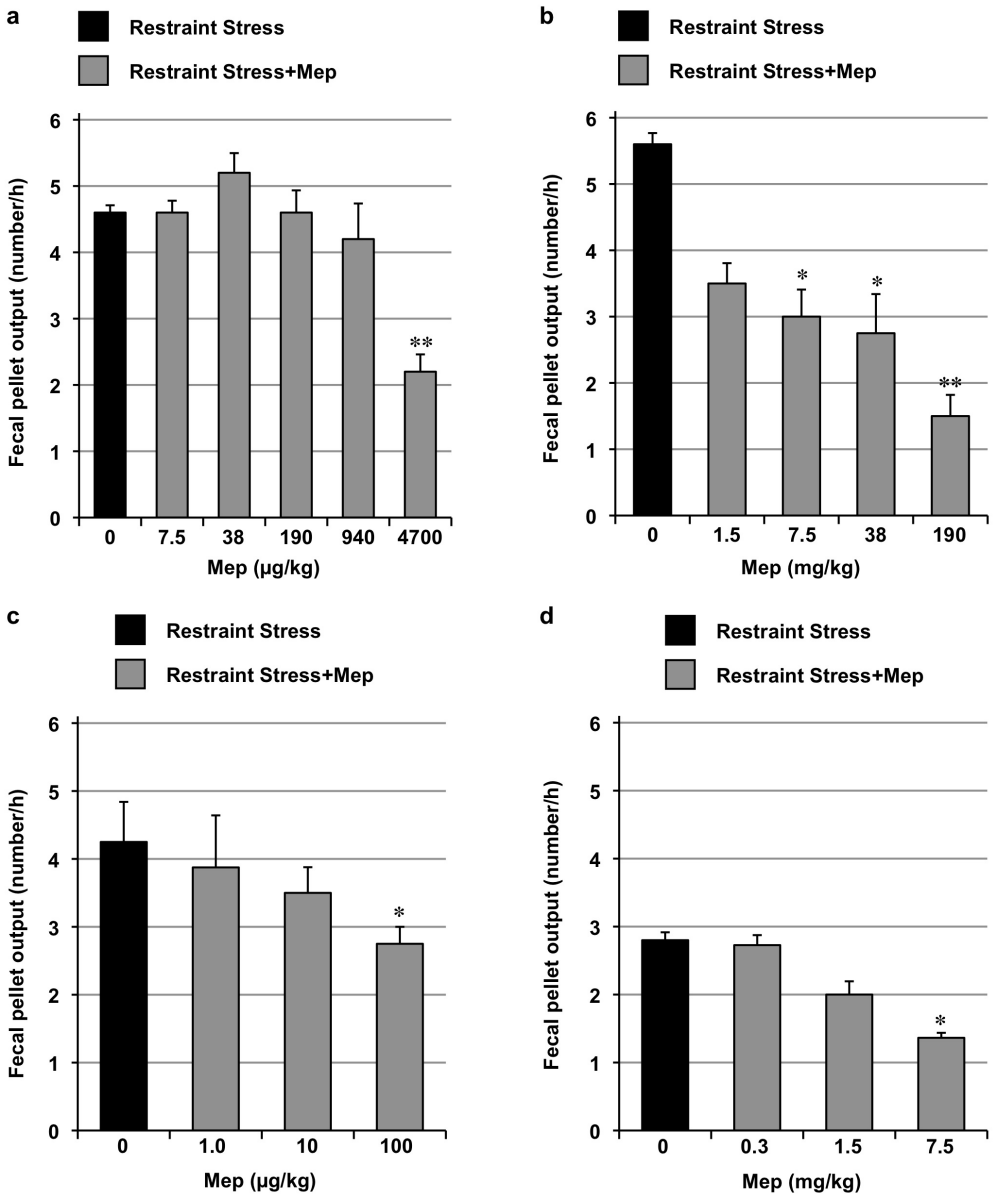
Effect of mepenzolate on fecal pellet output. Mice were administered indicated doses of mepenzolate intratracheally (a), orally (b), intravenously (c) or intrarectally (d). One hour later, mice were exposed to restraint stress. The number of fecal pellets excreted during the restraint stress period (1 h) was determined. Values represent mean ± S.E.M. (*n* = 4–15). * *P < 0.05*; ** *P < 0.01*.

Mice were subjected to restraint stress as a means to increase fecal pellet output. As shown in Fig. 7, mepenzolate administration suppressed fecal pellet output with respect to control (untreated) mice for each of the routes tested. Compared to the protective effects exerted by mepenzolate against PPE-induced pulmonary damage (Fig. 1), doses administered via the intratracheal administration route that were more than 100 times higher were required to affect fecal pellet output (Fig. 7a). In contrast, less than one hundredth the dose of mepenzolate required to provide a protective effect against lung damage significantly affected fecal pellet output when the oral administration route was used (Fig. 7b). As for the intravenous or intrarectal routes of administration, roughly similar doses of mepenzolate were required for both inhibition of fecal pellet output and protection against PPE-induced pulmonary damage (Figs. 3c, 5c, 7c and 7d). These results suggest that intratracheally administered mepenzolate could protect against PPE-induced pulmonary damage without affecting gut motility. Moreover, the results also suggest that orally administered mepenzolate affects gut motility directly (but not after absorption), because the dose required to suppress fecal pellet output was much lower compared to that required for other pharmacological effects.

**Figure 8:**
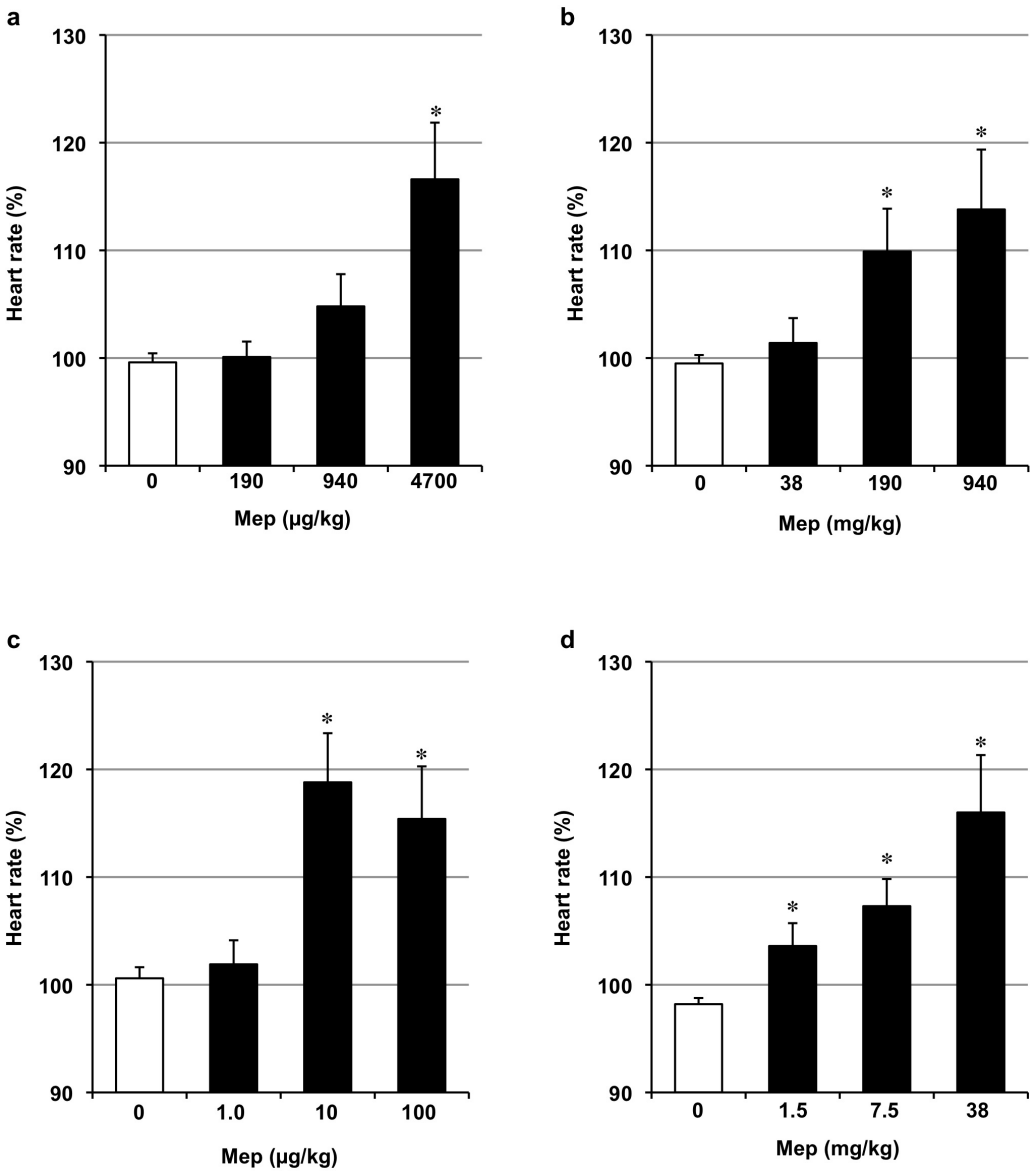
Effect of mepenzolate on heart rate. Mice were administered indicated doses of mepenzolate intratracheally (a), orally (b), intravenously (c) or intrarectally (d). The alteration of heart rate (beats per minute) by the mepenzolate administration was monitored as described in the Materials and Methods. Mepenzolate-dependent alteration of heart rate from the baseline to the peak is shown. Values represent mean ± S.E.M. (*n* = 3–7). * *P < 0.05*; ** *P < 0.01*.

Lastly, we examined the effect of mepenzolate on heart rate as measured by infrared sensor. As shown in Fig. 8a, intratracheally administered mepenzolate increased heart rate only at a dose that was much higher than that required to protect against PPE-induced pulmonary damage (Fig. 1c). On the other hand, the oral, intravenous or intrarectal routes of mepenzolate administration increased the heart rate at doses roughly similar to that required for pulmonary protection (Figs. 2c, 3c, 5c, 8b–d). These results suggest that intratracheally administered mepenzolate protects against PPE-induced pulmonary damage without affecting heart rate.

## Discussion

Since COPD is characterized by airflow limitation and abnormal inflammatory responses, a combination of anti-inflammatory drugs (such as steroids) and bronchodilators is the standard treatment regime^24^,^25^. Since mepenzolate has both anti-inflammatory and bronchodilatory activities, this drug may be beneficial for treating COPD without the concomitant use of other drugs. In particular, the anti-inflammatory effect of mepenzolate is an important property of this drug, because the inflammation associated with COPD tends to show resistance to steroid treatment; common steroids as such do not significantly modulate disease progression and mortality^5^,^6^,^7^. This insensitivity to steroids can be explained by the notion that steroids suppress the expression of pro-inflammatory genes via their action on histone deacetylase (HDAC) 2^26^,^27^. CS also inhibits the activity and expression of HDAC2^26^. On the other hand, mepenzolate can restore HDAC activity under inflammatory conditions^9^, which may explain its superior anti-inflammatory activity to steroids under these conditions (see below). In an animal model of elastase-induced lung inflammation and emphysema, we reported that steroids do not provide protective or therapeutic benefits against PPE-induced pulmonary emphysema, alterations of lung mechanics, or respiratory dysfunction^19^, whereas mepenzolate was effective against these disorders under the same experimental conditions^9^. Based on these results, we considered that mepenzolate could be therapeutically beneficial to treat COPD patients, which motivated us to examine here the effect of different routes of mepenzolate administration (intratracheal, oral, intravenous or intrarectal) on its beneficial effects (protection against PPE-induced pulmonary damage and bronchodilation) and adverse side-effects (alteration of gut motility and heart rate) in mice.

Intratracheally administered mepenzolate protected against PPE-induced pulmonary damage (inflammatory responses, pulmonary emphysema, alteration of lung mechanics and respiratory dysfunction) at a dose of 38 μg/kg and showed bronchodilation activity at a dose of 0.3 μg/kg, as reported recently^9^. We here found that this mode of administration required a much higher dose (4.7 mg/kg) to affect fecal pellet output and heart rate, thus demonstrating that intratracheally administered mepenzolate could suppress PPE-induced pulmonary damage and improve airflow limitation without affecting these other parameters, which is of particular clinical significance in terms of the use of this drug to treat COPD patients. This may be due to the fact that intratracheally administered mepenzolate is localized within the lung, in contrast to the other routes of administration studied. Furthermore, the lower dose of mepenzolate required for bronchodilation (compared to protection against PPE-induced pulmonary damage) suggests that intratracheally administered mepenzolate is localized within the bronchi rather than the alveoli, because such differences in dosage were not observed for the other forms of systemic administration.

We found here that the oral and intravenous routes of mepenzolate administration also protected against PPE-induced pulmonary damage and showed bronchodilatory activity. However, the improvement of respiratory function (FEV_0.05_/FVC) by mepenzolate was not statistically significant when the drug was administered via these routes. Compared to intravenous or intratracheal administration, much higher doses of mepenzolate were required to protect against PPE-induced pulmonary damage for the oral route of administration, suggesting that the efficiency of absorption into the circulation is very poor for administration via this route. It should be noted that mepenzolate achieved beneficial and adverse effects at roughly similar doses when administered orally or intravenously (except for the effect of orally administered mepenzolate on fecal pellet output). When the route of administration was intrarectal rather than oral, the effective dose of mepenzolate was decreased. However, as for the oral and intravenous routes of administration, intrarectally administered mepenzolate exerted both beneficial and adverse side-effects at roughly similar doses.

To determine the appropriate administration route of candidate drugs in a clinical setting, the most important factor is the balance between efficacy and safety. To estimate this factor in animals, the ratio between doses showing adverse effects and efficacy is useful. We calculated this index (Table 1) and results show the superiority of the pulmonary administration route for mepenzolate compared to other routes. The quality of life (QOL) of patients is also an important factor, for which the intravenous route of administration has a disadvantage. As well as oral administration, pulmonary administration (such as inhalation) would not overly affect the QOL of COPD patients given that most of these patients would already be required to take bronchodilators and/or steroids on a daily basis at home through inhalation.

On the other hand, one of the main advantages of the oral route of mepenzolate administration is that it already has regulatory approval, and most pre-clinical tests (such as toxicity and pharmacokinetic tests) could be omitted if the dose for a new indication (COPD) is less than that for the approved indication (gastrointestinal disorders). However, we found that the dose of orally administered drug required to protect against PPE-induced pulmonary damage was much higher than that at which fecal pellet output is affected, suggesting that the clinical dose of mepenzolate for the treatment of COPD would be higher than the already approved dosage. On the other hand, if mepenzolate is developed as a drug to be administered via the pulmonary route, although some pre-clinical tests (such as toxicity and pharmacokinetic tests) are required, other tests (such as genotoxicity tests) could be omitted. Furthermore, because the dose required to protect against PPE-induced pulmonary damage via the intratracheal route was much lower than the orally administered dose that affects fecal pellet output, it could be postulated that the clinical dose of mepenzolate required for the treatment of COPD patients may be lower than the already approved dose if this drug is developed as a drug to be administered intrapulmonary. This could decrease the risk of adverse effects in a clinical setting. In conclusion, we propose that the pulmonary administration of mepenzolate may be superior to other administration routes for the treatment of COPD.

## Methods

### Chemicals and animals

Mepenzolate, PPE and HPLC-grade acetonitrile were obtained from Sigma-Aldrich (St. Louis, MO). Novo-Heparin for injection was from Mochida Pharmaceutical Co. (Tokyo, Japan). Chloral hydrate was from Nacalai Tesque (Kyoto, Japan). Diff-Quik was from the Sysmex Co (Kobe, Japan). Sodium 1-propanesulfonate was from Tokyo Kasei Chemical Co (Tokyo, Japan). The Amicon utra-0.5 centrifugal filter unit was purchased from Merck Millipore (Billerica, MA). Formalin neutral buffer solution, potassium dihydrogen phosphate and methylcellulose were from WAKO Pure Chemicals (Tokyo, Japan). Mayer's hematoxylin, 1% eosin alcohol solution and malinol were from MUTO Pure Chemicals (Tokyo, Japan). ICR mice (4–6 weeks old, male) were purchased from Charles River (Yokohama, Japan). The experiments and procedures described here were carried out in accordance with the Guide for the Care and Use of Laboratory Animals as adopted and promulgated by the National Institutes of Health, and were approved by the Animal Care Committee of Keio University.

### Treatment of mice with PPE, CS and drugs

Mice maintained under anesthesia with chloral hydrate (500 mg/kg) were given one intratracheal administration of PPE (15 U/kg) and mepenzolate (various doses) in PBS (1 ml/kg) via micropipette. For control mice, PBS alone was administered by the same procedure.

ICR mice were exposed to CS by placing 15–20 mice in a chamber (volume, 45 L) connected to a CS-producing apparatus. Commercial non-filtered cigarettes (Peace®; Japan Tobacco Inc., Tokyo, Japan) that yielded 28 mg tar and 2.3 mg nicotine on a standard smoking regimen were used. Mice were exposed to the smoke of 2 cigarettes for 20 min, 3 times/day for 3 days. The apparatus was configured such that each cigarette was puffed 15 times over a 5 min period.

For the oral or intrarectal mode of administration, mepenzolate (various doses) in 1% methylcellulose was administered by sonde. For control mice, 1% methylcellulose alone was administered by the same procedure.

For the intravenous administration of mepenzolate, mice were maintained under anesthesia with chloral hydrate (500 mg/kg) and mepenzolate (various doses) in PBS was administered by syringe via a 26 G needle (TERUMO, Tokyo, Japan). For control mice, PBS alone was administered by the same procedure.

At day 0, the administration of mepenzolate was performed 1 h (intratracheal administration) or 0.5 h (other routes of administration) prior to the PPE administration or the CS exposure.

### Preparation of BALF and cell count method

BALF was collected by cannulating the trachea and lavaging the lung with 1 ml of sterile PBS containing 50 U/ml heparin (2 times). About 1.8 ml of BALF was routinely recovered from each animal. The total cell number was counted using a hemocytometer. Cells were stained with Diff-Quik reagents after centrifugation with Cytospin® 4 (Thermo Electron Corporation, Waltham, MA), and the ratio of number of neutrophils to total cell number was examined to determine the number of neutrophils.

### Histopathological analysis

Lung tissue samples were fixed in 10% formalin neutral buffer solution for 24 h at a pressure of 25 cmH_2_O, and then embedded in paraffin before being cut into 4 μm-thick sections. Sections were stained first with Mayer's hematoxylin and then with 1% eosin alcohol solution (H & E staining). Samples were mounted with malinol and inspected with the aid of an Olympus BX51 microscope (Tokyo, Japan).

To determine the MLI (an indicator of airspace enlargement), 20 lines (500 μm) were drawn randomly on the image of a section and intersection points with alveolar walls were counted to determine the MLI. This morphometric analysis was conducted by an investigator blinded to the study protocol.

### Measurement of lung mechanics, airway resistance and FEV_0.05_/FVC

Lung mechanics and airway resistance were monitored with a computer-controlled small-animal ventilator (FlexiVent, SCIREQ, Montreal, Canada), as described previously^18^,^19^. Mice were anesthetized with chloral hydrate (500 mg/kg), a tracheotomy was performed, and an 8 mm section of metallic tube was inserted into the trachea. Mice were mechanically ventilated at a rate of 150 breaths/min, using a tidal volume of 8.7 ml/kg and a positive end-expiratory pressure of 2–3 cmH_2_O.

Total respiratory system elastance and tissue elastance were measured by the snapshot and forced oscillation techniques, respectively. All data were analysed using FlexiVent software (version 5.3; SCIREQ, Montreal, Canada).

For measurement of methacholine-induced increases in airway resistance, mice were exposed to nebulized methacholine (1 mg/ml) five times for 20 sec with a 40 sec interval, and airway resistance was measured after each methacholine challenge by the snapshot technique. All data were analysed using the FlexiVent software.

Determination of the FEV0.05/FVC (forced expiratory volume in the first 0.05 seconds to forced vital capacity) ratio was performed with the same computer-controlled small-animal ventilator connected to a negative pressure reservoir (SCIREQ, Montreal, Canada), as described previously^18^,^19^. Mice were tracheotomised and ventilated as described above. The lung was inflated to 30 cmH_2_O over one second and held at this pressure. After 0.2 sec, the pinch valve (connected to ventilator) was closed and after 0.3 sec, the shutter valve (connected to negative pressure reservoir) was opened for exposure of the lung to the negative pressure. The negative pressure was held for 1.5 sec to ensure complete expiration. FEV0.05/FVC was determined using the FlexiVent software.

### Analysis of fecal pellet output

Mice were subjected to restraint stress by being placed individually into a 50 ml tube (Becton Dickinson, Franklin Lakes, NJ) for 1 h, as described previously^28^. These tubes are small enough to restrain a mouse so that it is able to breathe but unable to move freely. The number of fecal pellets excreted during the restraint stress period (1 h) was measured.

### Measurement of heart rate

Heart rate was measured with a MouseOx system (STARR Life Sciences Corp., Allison Park, PA), as described previously^29^. Mice were anesthetized with chloral hydrate (500 mg/kg) and the sensor was attached to the thigh. Heart rate was determined using MouseOx software (STARR Life Sciences Corp., Allison Park, PA).

### Determination of the level of mepenzolate in vivo

After administration of mepenzolate, blood samples (800 μl) were taken periodically into centrifuge tubes containing heparin (50 μl) and centrifuged immediately (1000 × g, 10 min) to obtain the sample. Whole lungs were taken from mepenzolate-treated mice, homogenised in sterile PBS containing 50 U/ml heparin, and centrifuged (14, 000 × g, 1 min) to obtain the sample. An aliquot (300 μl) of each sample was ultrafiltered with an Amicon utra-0.5 centrifugal filter to extract the mepenzolate. The filtrate was analysed by analytical HPLC with a reverse-phase column (TSKgel Super-ODS, 150 × 4.6 mm, 2 μm, Tosoh Co., Tokyo, Japan), Waters 2695 Alliance separation module, and a Waters 2996 photodiode array detector (Waters, Milford, MA). Solution containing 30% (v/v) acetonitrile and 14 mM potassium dihydrogen phosphate/sodium 1-propanesulfonate buffer was used at a flow rate of 0.3 ml/min. Detection was performed at an optical density of 220 nm.

### Statistical analysis

All values are expressed as the mean ± S.E.M. Two-way ANOVA followed by the Tukey test or the Student's *t*-test for unpaired results was used to evaluate differences between three or more groups or between two groups, respectively. Differences were considered to be significant for values of *P* < 0.05.

## Author Contributions

Conception and design: K.T. and T.M.; Analysis and interpretation: K.T., T.A., N.Y., D.K., Y.Y., H.Y., S.K. and T.I.; Drafting the manuscript for important intellectual content: K.T., T.A., N.Y., H.W., T.M., H.S. and T.M.

## Figures and Tables

**Table 1:** Efficacy versus toxicity ratio for different routes of mepenzolate administration

Administration route	Intratracheal	Oral	Intravenous	Intrarectal
**Efficacy**	38 μg/kg	190 mg/kg	10 μg/kg	1.5 mg/kg
**Toxicity**	4700 μg/kg	7.5 mg/kg	10 μg/kg	1.5 mg/kg
**Toxicity/Efficacy**	120	0.04	1	1

The effective dose (efficacy) was determined as the minimum dose required to significantly suppress the PPE-induced increase in MLI (Figs. 1c, 2c, 3c and 5c). The toxic dose (toxicity) was determined as the minimum dose required to significantly affect either fecal pellet output or heart rate (Figs. 7 and 8). The ratio of the toxic dose versus the effective dose for each route of administration is shown.
